# Droplet digital PCR as an alternative to FISH for MYCN amplification detection in human neuroblastoma FFPE samples

**DOI:** 10.1186/s12885-019-5306-0

**Published:** 2019-01-28

**Authors:** Dinesh Babu Somasundaram, Sheeja Aravindan, Zhongxin Yu, Muralidharan Jayaraman, Ngoc T. B. Tran, Shibo Li, Terence S. Herman, Natarajan Aravindan

**Affiliations:** 1 Departments of Radiation Oncology, University of Oklahoma Health Sciences Center, 940 Stanton L. Young Boulevard, Oklahoma City, OK 73104 USA; 2 Stephenson Cancer Center, Oklahoma City, OK USA; 3 Department of Pathology, University of Oklahoma Health Sciences Center, 940 Stanton L. Young Boulevard, BMSB 451, Oklahoma City, OK 73104 USA; 4 Department of Cell Biology, University of Oklahoma Health Sciences Center, 940 Stanton L. Young Boulevard, BMSB 553, Oklahoma City, OK 73104 USA; 5 Department of Pediatrics, University of Oklahoma Health Sciences Center, 1200 Children’s Ave. Ste 14000, Oklahoma City, OK 73104 USA; 6 Department of Anesthesiology, University of Oklahoma Health Sciences Center, 920 SL Young Blvd #1140, Oklahoma City, OK 73104-5036 USA

**Keywords:** Neuroblastoma, MYCN amplification, Immunohistochemistry, FISH, ddPCR

## Abstract

**Background:**

MYCN amplification directly correlates with the clinical course of neuroblastoma and poor patient survival, and serves as the most critical negative prognostic marker. Although fluorescence in situ hybridization (FISH) remains the gold standard for clinical diagnosis of MYCN status in neuroblastoma, its limitations warrant the identification of rapid, reliable, less technically challenging, and inexpensive alternate approaches.

**Methods:**

In the present study, we examined the concordance of droplet digital PCR (ddPCR, in combination with immunohistochemistry, IHC) with FISH for MYCN detection in a panel of formalin-fixed paraffin-embedded (FFPE) human neuroblastoma samples.

**Results:**

In 112 neuroblastoma cases, ddPCR analysis demonstrated a 96–100% concordance with FISH. Consistently, IHC grading revealed 92–100% concordance with FISH. Comparing ddPCR with IHC, we observed a concordance of 95–98%.

**Conclusions:**

The results demonstrate that MYCN amplification status in NB cases can be assessed with ddPCR, and suggest that ddPCR could be a technically less challenging method of detecting MYCN status in FFPE specimens. More importantly, these findings illustrate the concordance between FISH and ddPCR in the detection of MYCN status. Together, the results suggest that rapid, less technically demanding, and inexpensive ddPCR in conjunction with IHC could serve as an alternate approach to detect MYCN status in NB cases, with near-identical sensitivity to that of FISH.

## Background

Neuroblastoma (NB) is the most common cancer in infants less than one year old (28%) [1, 2], and accounts for about 6% of all cancers in children [3]. In the United States, the neuroblastoma incidence rate has remained constant at approximately 700 new cases per year [4], and heavily contributes to pediatric cancer deaths (9.1%) [3, 5, 6]. Although significant improvements in overall survival (OS, 40–65%) [5, 6] have been achieved for children with NB in the past thirty years, such OS data mask significant variability in outcomes for different risk groups. Although the patients who present with low-risk (stage 1 and 2) NB experience a complete cure, more than half of the patients with high-risk NB will relapse with hematogenous metastasis [7], despite intensive multimodal therapy [5, 6, 8–15]. A cure after relapse of progressive disease is extremely rare, with a 5-year OS of < 10% and, 2% long-term survival; compared with 65% in low/intermediate-risk disease (38–71% long-term survival) [5, 6, 8, 9, 11, 12, 14, 16]. High-risk disease is typically characterized by several genetic alterations that indicate and/or drive poor prognosis for patients with NB, including amplification of the MYCN oncogene.

MYCN (V-myc myelocytomatosis viral-related oncogene, neuroblastoma-derived [avian]) is a cellular proto-oncogene of the MYC family of transcription factors. MYCN maps to the short arm of chromosome 2 at band 2p24.3. MYCN amplification encoding the transcription factor N-MYC has been documented in most of the malignant neuroblastomas that cover about 20–25% of all NB [17]. MYCN amplification has also been associated with many chromosomal events, including loss of the distal short arm of 1p, aberration at 11q, and gain of 17q [18]. N-MYC acts as a transcription factor recognizing a consensus sequence (CACGTG) and can activate genes that affect cell growth and differentiation [19]. Thus, MYCN amplification is associated with advanced stage, rapid tumor progression, and poor prognosis [20, 21]. Less than 5% of patients with early disease showed MYCN amplification, compared with 30–40% of patients with advanced disease [22]. MYCN amplification values usually range between 50 and 100 fold, although much higher values have been reported. Since research has revealed the association of MYCN amplification with NB evolution [23, 24], independent from disease stage and age at diagnosis, MYCN amplification has been used as the biomarker for risk stratification [17, 25, 26]. Thus, assessment of MYCN amplification is essential for the diagnostic evaluation of patients with NB [27]. MYCN amplification status in NB must be assessed across all conditions (i.e., new diagnosis, prognosis, prospective and retrospective) and in immediate facilities, without any limitations.

Since the original discovery of MYCN amplification in a substantial subset of patients with NB [23], a number of methodologies, including southern blotting [28], polymerase chain reaction (PCR) [29], differential PCR [30], quantitative PCR (QPCR) [31], fluorescence in situ hybridization (FISH) [32]/direct FISH [33], interphase quantitative FISH (IQ-FISH) [34], and chromogenic in situ FISH (CISH) [35] have been validated as assessment methods. In addition, immunohistochemistry (IHC) can be a convenient and cost-effective approach. Earlier studies have indicated that N-myc protein expression could serve as one of the most unfavorable prognostic factors in NB patients [36, 37]. However, in addition to its technical limitations (e.g., quality of antibody, sensitivity, selectivity), N-myc expression as a stand-alone measure may not always translate to amplification status and could lead to equivocal outcomes.

MYCN detection by FISH is widely accepted and currently used in clinical settings. With the ability to demonstrate the state of amplification heterogeneity of the tumor cells and the nature of amplification units (double-minute chromosomes or homogeneously stained regions), detection of MYCN amplification with FISH remains a reliable method. Despite such benefits, FISH assay is subjective evaluation of images, technically demanding, extremely expensive, and requires good fluorescence scope and technical expertise [38]. Many diagnostic laboratories lack either the expertise or the facilities to perform the test. Even in ideal circumstances, the results are often difficult to interpret, requiring the scrutiny of large numbers of individual cells by a highly experienced diagnostician [39]. Also, studies have shown that despite for its high specificity, FISH assay exhibited extremely low (~ 58%) sensitivity. In addition, the technical difficulties in using FFPE specimens for FISH assay (probes ability to penetrate the tissue, high-level of auto-fluorescence, ghost nuclei, loss or weak signals etc.,) and the extremely low sensitivity in FFPE specimens remain the major limitations in using such collections. Other limitations are discussed in detail elsewhere [40]. It is important to develop rapid, reliable, and cost-effective alternative strategies to assess MYCN amplification in fresh, frozen, or formalin-fixed, paraffin-embedded (FFPE) NB samples. Accordingly, we investigated the potential advantages of using highly sensitive Droplet Digital PCR (ddPCR) [41] technology in conjunction with qualitative IHC to detect MYCN amplification in fresh, frozen, and FFPE NB samples, and compared it with the current standard of detection, FISH.

## Methods

We examined specimens from 116 cases of human NB. Specimens were collected from our institutional (University of Oklahoma Health Sciences, OUHSC) pediatrics pathology collection (82 specimens), the Oregon Health and Science University Biospecimen core (24 specimens) and the NIH-NCI Cooperative Human Tissue Network (CHTN, 10 specimens). All protocols were approved by the University of Oklahoma Health Sciences Center Institutional Review Board with permission for the research use of de-identified specimens. All experiments were performed in accordance with the University of Oklahoma Health Sciences Institutional Review Board guidelines and regulations for the protection of human subjects. Hematoxylin-eosin stained sections were examined by a pediatric pathologist. Only cases with sufficient percent tumor (and minimal necrosis) and adequate tumor volume for multiple assays were included. On this basis, four specimens were excluded. The results were computed for a total of 112 samples, of which 79 specimens had known MYCN status, as assessed in clinics using FISH analysis.

### MYCN amplification detection by FISH

**Fig. 1:**
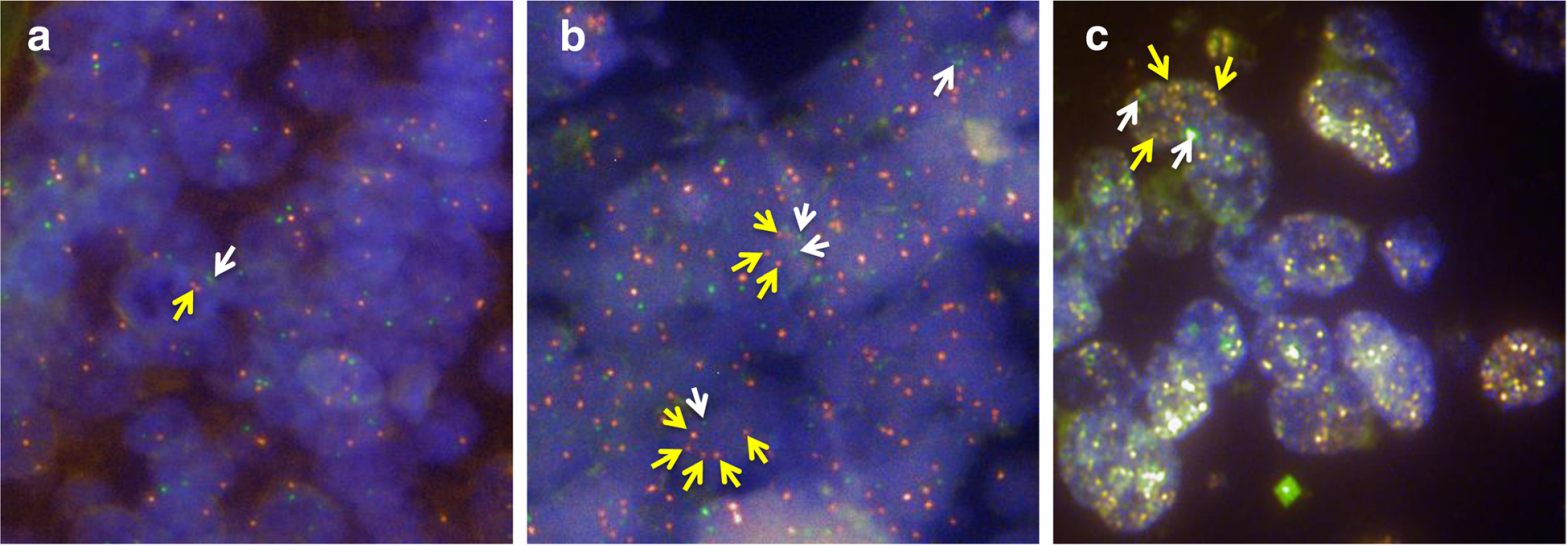
Fluorescence in situ hybridization (FISH) for MYCN amplification in human NB specimens. Representative microphotographs of MYCN FISH analysis showing (**a**) negative amplification (non-amplified) of human MYCN gene with the ratio of MYCN (red signals, indicated by yellow arrowheads) to AFF3 (green signals, indicated by white arrowheads) obviously 1 (2R2G), (**b**) positive amplification of human MYCN gene with amplified signal of 2 + R2G, and (**c**) positive MYCN amplification signal appearing as a homogenously stained region and/or double minutes containing numerous signals

For FISH analysis, the tumor region in 4-μm-thick FFPE sections was selected by the pathologist. For MYCN amplification, Kreatech™ MYCN (2p24) / AFF3 (2q11) FISH probe (Leica Biosystems Inc., Buffalo Grove, IL, USA) was used. MYCN Amplification at region 2p24 will show several red signals compared with the control AFF3 (2q11) region, which will provide two signals (Fig. 1). FISH assays were performed in the Cytogenetic Molecular division of the OUHSC Pediatrics Clinical Genetics Core and in the Tissue Pathology Core of the Stephenson Cancer Center, following standard protocols. Ten specimens, including four with known MYCN status (two amplified and two non-amplified; for assay controls) and six with unknown status were independently assayed in the two facilities. The FISH signals were independently evaluated by two investigators (NA, DS) and validated by a pediatric pathologist (ZY).

### N-myc expression detection by IHC

**Fig. 2:**
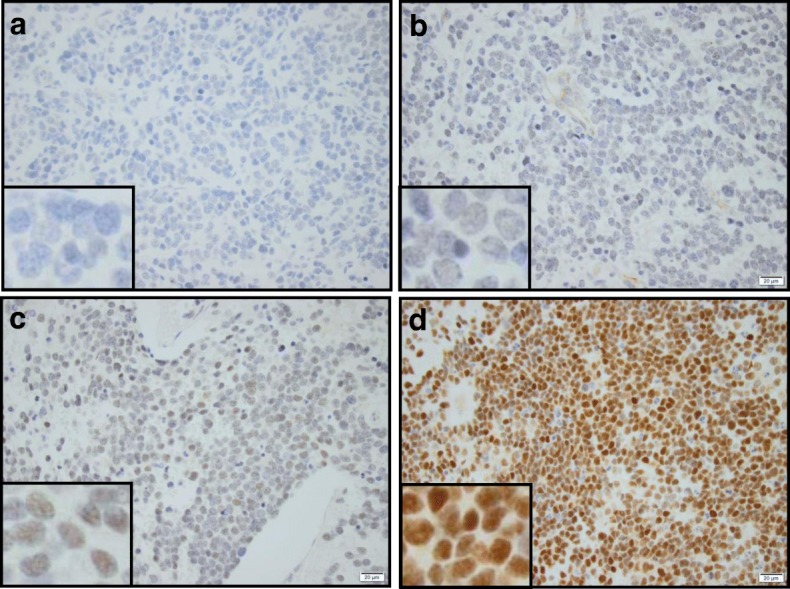
Immunohistochemistry for MYCN protein expression in human neuroblastomas. Representative microphotographs of MYCN IHC staining showing (**a**) completely negative (IHC0), (**b**) weak/faint nuclear positivity (IHC1+), (**c**) moderate nuclear positivity (IHC2+), and, (**d**) strong nuclear immunoreactivity (IHC3+) in FFPE sections from NB cases. Insert: Representative staining patterns shown in 40x magnification

All tissue section processing and immunohistochemistry (IHC) was performed in the Tissue Pathology Core of the Stephenson Cancer Center, as described in our earlier studies [42–44]. Mouse monoclonal N-myc antibody raised against human N-myc mapping to 2p24.3 (Santa Cruz Biotechnology Inc., Dallas, TX) was used. N-myc IHC was performed utilizing an automated IHC machine (Leica Bond III), according to the manufacturer’s protocol, using the Bond™ Polymer Refine detection system. A peroxidase-diaminobenxidine visualization process, which gave positive immunoreactivity a brown color, was employed. Appropriate tissue histology controls stained with hematoxylin-eosin stain and negative controls with no primary antibody were examined in parallel. The slides were digitally scanned into virtual slides using an Aperio Scan Scope (Aperio Technologies, Inc., Buffalo Grove, IL, USA) slide scanner at 20x magnification. The whole slide images were then group-analyzed for N-myc -specific positivity using Aperio image analysis and quantification software (Aperial Tool Box) with the appropriate algorithms for IHC. Automated strong nuclear positivity was quantified and the metadata were exported to Excel. N-myc expression was independently graded by two pathologists (ZY, NTT) who were blinded to FISH and ddPCR status. Scores for N-myc immunoreactivity in IHC staining were graded on a scale of 0–3 (0 = negative, 1 = weak, 2 = moderate, 3 = strong; Fig. 2). For inter-assay crisscross analysis, IHC grading results were further computed to fit the criteria, true positive vs. true negative expression. For this, grading scales of 2 and 3 are regarded as ‘positive’ and scale 1 and 0 as ‘negative’.

### MYCN amplification by ddPCR

**Fig. 3:**
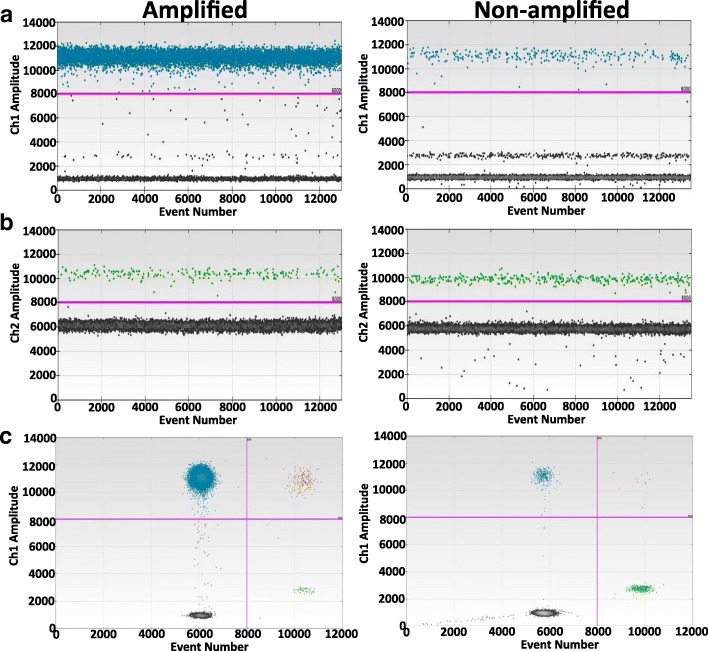
Analysis of MYCN amplification by droplet digital PCR in human NB specimens. Representative one dimensional ddPCR plots for (**a**) MYCN and (**b**) reference gene RNAseP showing side-by-side comparison of MYCN copy number variations in MYCN amplified and non-amplified NB cases. MYCN was read in blue (FAM) channel, while RNASeP was read in green (HEX) channel. Each point represents a single droplet, which is scored as positive (colored and above the threshold intensity, as indicated by the pink line) or negative (grey, below the threshold line), depending on the fluorescent amplitude. (**c**) Representative (from amplified and non-amplified cases) two-dimensional scatter plots constructed with overlaid ddPCR data of the reference RNAseP (HEX) and MYCN (FAM) showing droplets containing no template (lower left, black), droplets containing only MYCN template (upper left, blue), droplets containing only reference RNAseP template (lower right, green), and droplets containing both MYCN and RNAseP templates (upper right, brown)

For DNA isolation, ten 6-μM-thick sections were cut from each FFPE block and were collected in DNAse-free sterile microcentrifuge tubes. Genomic DNA was extracted using a HighPrep™ FFPE Tissue DNA Kit (MagBio, Gaithersburg, MD) according to the manufacturer’s protocol. The quality and the quantity of the isolated DNA were determined using our routine laboratory protocols [45]. MYCN copy number variations were assessed using TaqMan Copy Number (Hs00658058_cn, ThermoFisher Scientific, Waltham, MA) assay. RNaseP (4,403,326, ThermoFisher) assay was used for reference gene. For ddPCR, the HINDIII-digested DNA (5 ng) was subjected to PCR (final reaction volume of 20 μL) utilizing ddPCR™ Supermix for probes (BioRad, Hercules, CA). To generate droplets, individual reaction mixtures were then loaded into a DG8 cartridge (Bio-Rad) with 70 μL of droplet generation oil. The droplets from each well were transferred into a 96-well PCR plate, heat-sealed, and subjected to PCR: 95 °C for 10 min, followed by 40 cycles of 94 °C for 30 s, 60 °C for 1 min, and 98 °C for 10 min. The droplets of each well were then analyzed in a QX100 droplet reader (Bio-Rad) and were quantified using target DNA. The outcome data were analyzed using QuantaSoft version 1.7.4.0917 (Bio-Rad), and the copy number variation was determined (Fig. 3).

### Statistical analysis

All statistical analyses and graph plots were performed using GraphPad Prism software. Correlations between each assay were evaluated using nonlinear regression analysis, and the goodness of fit was calculated. Nonlinear regression analysis fits our model, which offers more options such as, the ability to compare two models (ddPCR vs FISH; ddPCR vs IHC; IHC vs FISH), apply weighting, automatically exclude outliers and perform normality tests on the residuals. In this study, for specimens with known FISH status (*n = 79*) the goodness of fit was calculated independently for ddPCR or IHC against FISH data. For the specimens with unknown FISH status (*n = 33*) the goodness of fit was calculated independently for ddPCR and FISH against IHC data. In each case, the quantification of the goodness-of-fit was presented as R square.

## Results

**Fig. 4:**
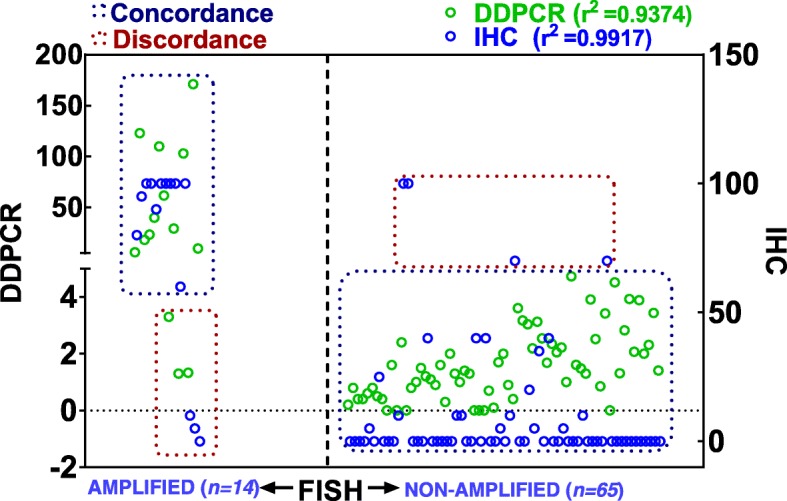
Comparison of ddPCR and IHC MYCN results with FISH data from the FFPE tissue samples from 79 neuroblastoma cases. Interleaved scatter-plot showing concordance (and discordance) in MYCN amplification status assessment by ddPCR and IHC compared with FISH analysis. A total of 79 neuroblastoma cases with known MYCN status (14 amplified and 65 non-amplified) assessed by FISH as a part of clinical diagnosis were included in the analysis

Seventy-nine specimens had known MYCN status per FISH analyses (*n* = 14, MYCN-amplified; *n* = 65, non-amplified). In these 79 specimens with known MYCN status, ddPCR demonstrated MYCN amplification in 11 specimens and no amplification in 68 specimens (Table 1, Fig. 4). Compared with FISH data, ddPCR results had 79% concordance (11/14) for amplified samples and 100% concordance (65/65) for non-amplified samples (Table 1, Fig. 4). Computing together the amplified and non-amplified cases, ddPCR results showed outstanding agreement (96.2% concordance and 3.8% discordance) with the FISH analysis (Table 1). Conversely, N-myc IHC coupled with pathologists’ grading revealed N-myc expression in 15 specimens and no expression in 64 specimens. Compared with FISH data, MYCN IHC results had concordance of 73% (11/15) for positive N-myc expression and 95.3% (61/64) concordance for negative N-myc expression (Table 1, Fig. 4). Overall, IHC results showed excellent agreement (91.2% concordance) with FISH. More importantly, comparative analysis between ddPCR and IHC data analysis revealed 100% concordance for MYCN-amplified cases (Table 1, Fig. 4). Conversely, we observed a concordance of about 94.1% (64/68) for non-amplified cases with ddPCR results (Table 1). For all 79 cases, there was a 94.9% concordance between ddPCR and IHC. Together, the ddPCR results coupled with IHC data corroborated with the known FISH status and strongly suggest that ddPCR and IHC could serve as an alternative to FISH, particularly for FFPE specimens.

**Table 1:** Inter-assay concordance analysis of human MYCN status determined by ddPCR, IHC (N-myc) and FISH in human neuroblastoma

DD-PCR	FISH Amplified	FISH Non-amplified	Concordance by DDPCR	Discordance by DDPCR
Non-amplified (*n = 68)*	3	65	(65/65)100%	(0/65)0%
Amplified (*n = 11)*	11	0	(11/14)79%	(3/14)21%
IHC	FISH Amplified	FISH Non-amplified	Concordance by IHC	Discordance by IHC
Negative (*n = 64)*	3	61	(61/64)95.3%	(3/64)4.7%
Positive (*n = 15)*	11	4	(11/15)73%	(4/15)27%
DD-PCR	IHC Positive	IHC Negative	Concordance by DDPCR	Discordance by DDPCR
Non-amplified (*n = 68)*	4	64	(64/68)94.1%	(4/68)5.9%
Amplified (*n = 11)*	11	0	(11/11)100%	(0/11)0%

A total of *79 selected cases with known MYCN status* (14 amplified and 65 non-amplified) assessed by FISH as a part of clinical diagnosis were included in the analysis. Number of cases and percent measures of concordance in *FISH* vs *ddPCR*, *IHC* vs. *FISH*, and *ddPCR* vs. *IHC* are presented for amplified and non-amplified cases

**Fig. 5:**
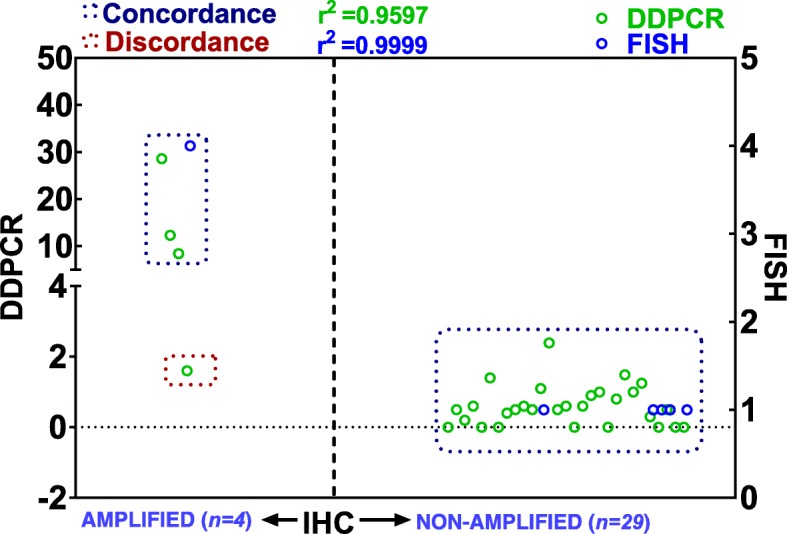
Inter-comparison of MYCN amplification status data from ddPCR, IHC, and FISH analyses of FFPE tissue samples from 33 NB cases. Interleaved scatter-plot showing concordance (and discordance) levels in MYCN amplification status measures between ddPCR, IHC, and FISH analyses. A total of 33 neuroblastoma cases with unknown MYCN status were included in the analysis. FISH was performed on 10 cases, four with known MYCN status (two amplified and two non-amplified) and six from the cohort of unknown status

To substantiate the benefits of using ddPCR in conjunction with IHC for MYCN amplification detection, we investigated the feasibility of assessment in a cohort of 33 NB cases with unknown MYCN status. First, as a fail-proof measure, we performed FISH on 10 cases, four with known MYCN status (two amplified and two non-amplified) and six from the cohort of unknown status. Since it is extremely challenging to perform FISH in FFPE sections, particularly in stored sections, and often produced equivocal results, two independent core facilities performed FISH with the same set of slides. The FISH analysis for the cases with known status yielded consistent results from both facilities, and served as the positive and negative controls for the assay (Fig. 1). Of the six cases with unknown status, FISH analysis revealed MYCN amplification in one specimen and no amplification in the remaining 5 specimens. ddPCR analysis of all 33 cases showed three cases with MYCN amplification and 30 cases without amplification (Fig. 5). In addition, IHC grading analysis revealed positive expression of N-myc in four cases and negative expression in 29 cases. Compared with ddPCR data, IHC had 96.7% concordance (29/30) for non-amplified samples and 100% concordance (3/3) for amplified cases (Table 2, Fig. 5). More importantly, comparative analysis between all three assay platforms demonstrated perfect concordance (100%) of FISH results with both the ddPCR and IHC analysis (Table 2, Fig. 5).

**Table 2:** Inter-assay concordance analysis of human MYCN status determined by ddPCR and IHC (N-myc) in human NB

DD-PCR	IHC Positive	IHC Negative	Concordance by DDPCR	Discordance by DDPCR
Non-amplified (*n = 30)*	1	29	(29/30)96.7%	(1/30)3.3%
Amplified (*n = 3)*	3	0	(3/3)100%	(0/3)0%

A total of ***33 blinded cases with unknown MYCN status*** were included in the analysis. Number of cases and percent measures of concordance in ***ddPCR*** **vs.** ***IHC*** is presented for amplified and non-amplified cases

## Discussion

Digital droplet PCR is a promising platform for high throughput assessment and quantitation of the targeted copy number variation. In this study, we demonstrate that the ddPCR platform is comparable to traditional FISH method for MYCN gene amplification in NB. In about 20–25% of neuroblastomas, poor prognosis has been directly correlated with MYCN oncogene [46] amplification, which has been shown to orchestrate rapid progression and therapy resistance [20, 47–49]. Since MYCN amplification is directly correlated with aggressive clinical course of NB and poor patient survival, it has been recognized as the most critical negative prognostic marker. To that end, MYCN amplification status currently guides the therapeutic strategy in children with otherwise favorable prognostic indicators [50]. Due to its significance, reliable laboratory data for evaluating MYCN status are essential. Currently, FISH analysis (MYCN status at the level of DNA) and IHC (protein expression) are used in clinical diagnosis. Although IHC is easy to perform, rapid, and relatively cost-effective, limitations, including the sensitivity, specificity and functional relevance, indicate that IHC should be considered only as a secondary and/or confirmatory approach. To date, FISH analysis remains the gold standard for assessing MYCN amplification status in NB. However, it is technically demanding, very expensive, and requires specific equipment and expertise [38, 39]. Moreover, it is highly challenging to perform FISH assays in FFPE sections. Such limitations [40] highlight the need for the development and use of less expensive, less technically demanding, easily accessible, and rapid methods for MYCN detection in NB that could produce near-identical sensitivity to that of FISH.

The results presented here show that detection of MYCN amplification by ddPCR will fill this gap with 96–100% concordance and could serve as an alternative to FISH analysis. However, the present study did not compare the sensitivity and specificity of FISH and ddPCR protocols in the NB setting. The results presented here, for the first time, demonstrate that ddPCR in conjunction with IHC grading could serve as an alternative to detect MYCN amplification status in NB specimens. Our experience in detecting MYCN using FFPE specimens clearly shows that ddPCR is less challenging than FISH analysis.

ddPCR has been adopted in the determination of copy number variation an array of tumor systems, including breast cancer [51], gastric cancer [52], and lung cancer [53]. Researchers have reported the benefit of using ddPCR in FFPE specimens [54, 55]. The results of the present study, for the first time, indicated the potential use of ddPCR in the detection of MYCN amplification status in NB cases and further recognized the reliability and feasibility of ddPCR in determining MYCN status from FFPE specimens. High-throughput ddPCR yields absolute quantitation of DNA copy number with an immediate utility to determine copy number variation and detect rare alleles and circulating DNA. Compared with FISH, ddPCR offers high-throughput analysis with simple workflow [56], and is cost-effective (~$30/rxn vs. a minimum of $300 for FISH). Moreover, ddPCR is highly sensitive, provides absolute copy number variation, utilizes low DNA concentration, allows absolute quantitation, and is a rapid process (8-10 h/16 samples from DNA-isolation to ddPCR readout vs. days for FISH). ddPCR sample analysis time frame could be extrapolated for 96 samples with added time for additional sample DNA isolation. Also, ddPCR analysis demands basic technical expertise compared to FISH assay that requires scrutiny of large numbers of individual cells by a highly experienced diagnostician. Further, the technical difficulties (probes ability to penetrate the tissue, high-level of auto-fluorescence, ghost nuclei, loss or weak signals etc.,) in FISH for handling FFPE specimens are entirely eluded in ddPCR (directly utilize DNA) and hence offers relatively high sensitivity. Although the FISH to ddPCR discordance rate observed here was negligible (0–5%), many factors could be responsible and should be considered limitations for ddPCR. These factors include the variability in the quality of DNA extracted from FFPE samples, availability of tumor tissues in the sections, tumor to necrosis ratio, and technical issues in droplet generation. To that end, ddPCR includes a multi-step (requirement to generate droplets) procedure and the specific target is limited. Furthermore, this study did not include the microdissection method to obtain cancer cells from the FFPE samples for ddPCR processing. Despite, this method is highly efficient as it enables objective evaluation with the provision of numerical values when compared to conventional methods that depend on the subjective evaluation of images. In addition to the limitations of ddPCR discussed above, authors acknowledge that ddPCR requires designated space to minimize the risk of contamination that could limit its adoption in some clinical environments. Also on a minor note, at least in the present study, ddPCR utilizes relatively more tissue (10 × 6 μM sections) while for FISH analysis only two 4 μM sections is used. However owing to its advantages ddPCR platform could serve as a promising alternative for the conventional methods. Also, ddPCR platform will be highly useful for retrospective studies that involve analysis of samples in hundreds.

Studies have clearly affirmed that N-myc protein expression could serve as one of the most unfavorable prognostic factors in NB patients [36, 37]. However, the discordance between FISH and IHC or ddPCR and IHC could be attributable to factors including variability in tissue fixation/processing, variable sensitivity/specificity of commercially available antibodies, variations in grading criteria and inter-observer variability in data interpretation [57]. Furthermore, IHC measures the amount of accumulated N-myc protein that could greatly depend on the transcriptional and post-transcriptional mechanisms existing in some tumors. The results presented in this study affirms the prognostic significance of N-myc expression and aligns with the earlier COG study [37]. To that end, the claim is that N-myc expression with IHC could serve as an additional validation to define MYCN status when used in conjunction with ddPCR.

In conclusion, the results of the present study showed that MYCN amplification status in NB cases could be assessed by relatively cost-effective, rapid, feasible, and reliable high-throughput ddPCR. Further, the results indicated that ddPCR could serve as a less technically challenging method to detect MYCN status in FFPE NB specimens. These findings revealed the concordance between FISH and ddPCR analyses in the detection of MYCN amplification status in FFPE NB specimens. Overall, the results presented here suggest that ddPCR, in conjunction with IHC, could serve as an alternate approach to detect MYCN status in NB cases, with near-identical sensitivity to FISH. This approach is highly beneficial in two settings (i) in places where there is a shortage of expertise, instrumentation and funds to use FISH and (ii) when there is a retrospective study involving hundreds and thousands of cases to investigate. Furthermore, the ddPCR approach has significant advantage over FISH for the FFPE specimens.

## Abbreviations

CISH: Chromogenic in situ FISH
ddPCR: Droplet Digital PCR
FFPE: Formalin-fixed, paraffin-embedded
FISH: Fluorescence in situ hybridization
IHC: Immunohistochemistry
IQ-FISH: Interphase quantitative FISH
MYCN: V-myc myelocytomatosis viral-related oncogene, neuroblastoma-derived [avian]
NB: Neuroblastoma
N-myc: Proto-oncogene protein, basic helix-loop-helix protein 37 (bHLHe37), protein encoded by the MYCN gene.
OS: Overall survival
PCR: Polymerase chain reaction
QPCR: Quantitative PCR
